# Atrial Flutter and Left Hemidiaphragmatic Paralysis in the Setting of Lyme Disease

**DOI:** 10.7759/cureus.37374

**Published:** 2023-04-10

**Authors:** Jeremy Palmer, Kearn Ghuman, Kiran Suhail, Nancy D Nagib

**Affiliations:** 1 Family Medicine, Wellspan Health York Hospital, York, USA; 2 Family Medicine, Fauquier Health, Warrenton, USA; 3 Family Medicine, Edward Via College of Osteopathic Medicine, Blacksburg, USA; 4 Family Medicine, WellSpan Health York Hospital, York, USA

**Keywords:** infectious disease medicine, outpatient family medicine, neurological lyme, atrial flutter, unilateral diaphragmatic paralysis, lyme's disease

## Abstract

Lyme disease, caused by a tick-borne spirochete, *Borrelia burgdorferi, *is the most common vector-borne disease in the United States. Clinical manifestations can include erythema migrans, carditis, facial nerve palsy, or arthritis. A rare complication of Lyme disease is hemidiaphragmatic paralysis. The first case of this complication was documented in 1986, and since then, there have been 16 case reports associating hemidiaphragmatic paralysis with Lyme disease. This is a case of a patient found to be in atrial flutter likely resulting from left hemidiaphragmatic paralysis as a complication of Lyme disease. The patient was a 49-year-old male recently diagnosed with Lyme disease who was treated with a 10-day course of doxycycline and who presented with dyspnea and chest pain. He appeared in acute distress with tachypnea and tachycardia to 169 beats/minute but was not hypoxic. Electrocardiogram (EKG) showed atrial flutter with a rapid ventricular response (RVR). The patient was sent to the emergency department and was treated with intravenous (IV) metoprolol, followed by an IV diltiazem drip, and ultimately converted to normal sinus rhythm. Chest X-ray demonstrated an elevated left hemidiaphragm. Due to concern for Lyme carditis causing tachyarrhythmia, the patient was started on IV ceftriaxone 2 g daily. A transthoracic echocardiogram showed no valvular abnormalities and a normal ejection fraction, thus indicating a low likelihood of carditis. The patient was transitioned to oral doxycycline for an additional 17 days. During the hospital course, a fluoroscopic chest sniff test confirmed the left hemidiaphragmatic paralysis. A chest X-ray completed after two months showed persistent elevation of the left hemidiaphragm and the patient continued to have mild dyspnea. The main lesson from this case is to consider hemidiaphragmatic paralysis as a possible complication of Lyme disease.

## Introduction

Lyme disease is a multisystem tick-borne infectious disease, caused by the spirochete *Borrelia burgdorferi*. According to the Centers for Disease Control and Prevention (CDC), approximately 476,000 Americans are diagnosed and treated for Lyme disease each year [1]. Complications observed from Lyme disease can include erythema migrans, carditis, facial nerve palsy, or arthritis. The most common signs and symptoms of Lyme carditis include heart palpitations and an irregular heartbeat [1]. Diagnosis of the arrhythmia is typically made with a 12-lead electrocardiogram (EKG). While an atrioventricular block is the most frequent manifestation of Lyme carditis, approaching arrhythmias with caution in the setting of Lyme disease is important, as many of our traditional treatment approaches for arrhythmias would be considered unsafe. For example, electrical cardioversion in the setting of an inflamed myocardium could be proarrhythmogenic and fatal [2].

Lyme disease has also been shown to cause various neurologic sequelae, as it can affect the central and peripheral nervous systems with presentations such as meningitis, meningoencephalitis, cranial neuropathy, myelitis, and radiculopathy [3]. Another common finding of Lyme disease is paralysis of the facial nerve presenting as a facial droop. This was reported in about 3% of the population [4]. However, paralysis of the diaphragm is a rare complication. The phrenic nerve of the diaphragm includes the C3 through C5 nerve supply. The first known case of diaphragmatic paralysis as a complication of Lyme disease was reported in 1986 [5]. This case is unique because the patient presented with cardiopulmonary abnormalities that are not typical of Lyme disease. This case demonstrates the need for more research regarding the rare complications caused by Lyme disease.

## Case presentation

Patient information

The patient is a 49-year-old male diagnosed with Lyme disease one month earlier. He is an avid hiker and frequented trails in Pennsylvania where Lyme is endemic. He presented at that time with fatigue and was subsequently positive for Lyme antibody and Western blot tests. He then received a 10-day course of doxycycline. He overall felt better after taking the antibiotics; however, he presented to the office one month later with a complaint of shortness of breath for the past two weeks. The patient is a nonsmoker with no history of lung disease or cardiovascular risks. The patient had no other relevant family history, psycho-social history, or past interventions.

Clinical findings

On the physical exam, the patient presented as a well-nourished, well-developed Caucasian male. He was alert and oriented to person, place, and time. On presentation, he was in acute distress with tachypnea and tachycardia. He presented with a heart rate of 169 beats per minute. His blood pressure was 160/98 mmHg. On his cardiac exam, no murmurs, rubs, or gallops were appreciated. Lungs were clear on auscultation bilaterally.

Timeline

**Figure 1:**
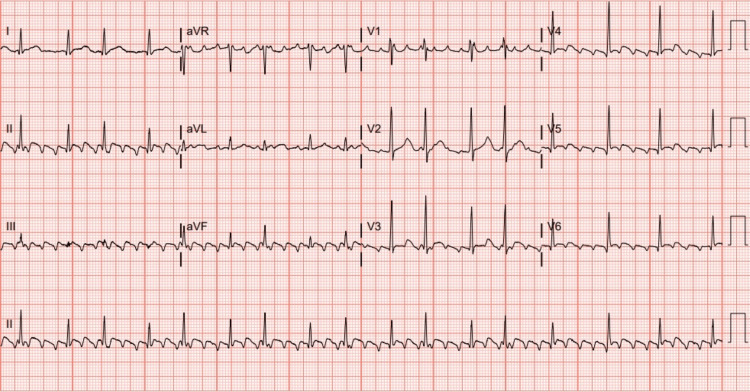
Initial EKG showing atrial flutter

**Figure 2:**
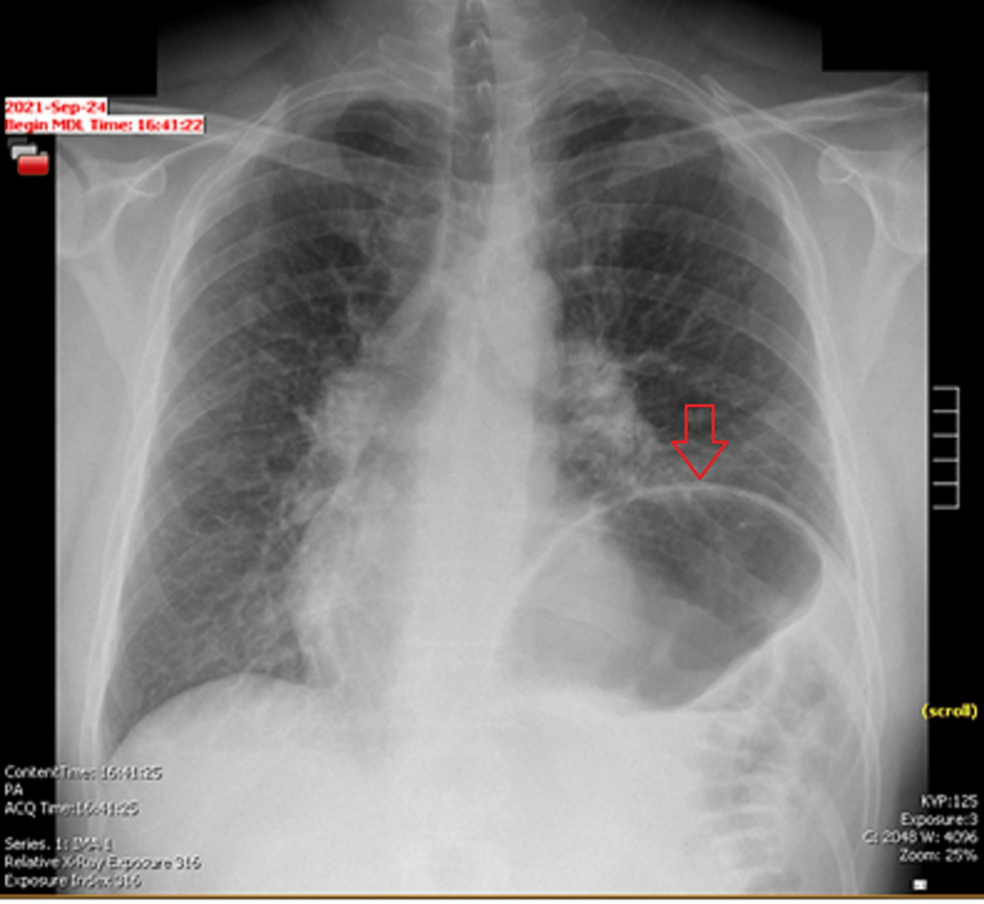
Initial chest X-ray showing left hemidiaphragmatic paralysis

The initial EKG (Figure 1) at the office showed atrial flutter with a rapid ventricular rate and was urgently sent to the emergency department. The patient was admitted to the hospital for further evaluation and treatment. On hospital day 1, the patient was initially trialed on intravenous (IV) metoprolol with no success in rate control. He was then transitioned to an IV diltiazem drip, which decreased his heart rate to below 110 beats per minute on hospital day 1. The patient spontaneously converted to normal sinus rhythm while on the IV diltiazem drip. Due to concern for Lyme carditis, the patient was started on IV ceftriaxone 2 g daily. A fluoroscopic chest sniff test was performed due to the elevated left hemidiaphragm seen on the initial chest X-ray (Figure 2). The results of the sniff test confirmed the finding of left hemidiaphragmatic paralysis (Video 1). On hospital day 4, a transthoracic echocardiogram (TTE) was completed to check for Lyme carditis. The patient was then transitioned to oral doxycycline 100 mg twice a day for an additional 17 days. He was discharged on hospital day 5 to complete the remaining course of antibiotics at home.

**Video 1:** Sniff test showing left hemidiaphragmatic paralysis

Diagnostic assessment

Diagnoses that were considered included pulmonary embolism, Lyme carditis, and post-treatment Lyme disease syndrome. As stated above, multiple diagnostic tests were conducted for the patient, including EKG, chest X-ray, CT angiography (CTA) lungs, fluoroscopic chest sniff test, and TTE. The EKG (Figure 1) was consistent with atrial flutter with RVR. The chest X-ray (Figure 2) showed an elevated left hemidiaphragm with left basilar atelectasis and a prominent gastric bubble; no cardiac abnormalities were noted. The CTA showed no acute abnormalities and ruled out pulmonary embolism. The findings of the TTE showed a low likelihood of Lyme carditis. The diagnosis of diaphragmatic paralysis was confirmed with the paradoxical movement of the left hemidiaphragm on the fluoroscopic chest sniff test. The patient’s labs were drawn which were overall reassuring (Table 1). While at the hospital, the patient did not face any diagnostic challenges.

**Table 1:** Laboratory results on initial evaluation BUN: blood urea nitrogen; eGFR: estimated glomerular filtration rate; BNP: brain natriuretic peptide; TSH: thyroid-stimulating hormone

		Reference Range
Complete Blood Count		
WBC	5.8 K/mcL	4.0 - 11.0 K/mcL
Hb	14.1 g/dL	13.0 - 17.3 g/dL
Plts	177 K/mcL	140 - 400 K/mcL
Metabolic Panel		
Glu	120 mg/dL	70 - 99 mg/dL
Na	138 mEq/L	135 - 145 mmol/L
K	3.7 mmol/L	3.5 - 5.3 mmol/L
CO2	25 mEq/L	21 - 31 mmol/L
BUN	18 mg/dL	7 - 25 mg/dL
Creatinine	1.02 mg/dL	0.70 - 1.30 mg/dL
eGFR	70.6 mL/min/1.73m*2	>=60.0 mL/min/1.73m*2
Mg	1.9 mg/dL	1.7 - 2.6 mg/dL
Cardiac Panel		
Troponin	<0.03 ng/mL	<0.03 ng/mL
BNP	81 pg/mL	<=100 pg/mL
Thyroid Panel		
TSH	1.70 mIU/L	0.30 - 5.00 mcIU/mL

Therapeutic intervention

Upon the patient’s hospitalization, he received four doses of IV metoprolol 5 mg and metoprolol tartrate 50 mg PO without resolution of his atrial flutter. He then received one dose of IV diltiazem 10 mg and following this was started on a diltiazem drip. The patient then spontaneously converted to normal sinus rhythm confirmed by an EKG. The patient did not require cardioversion. For concerns of infectious Lyme carditis, the patient was on IV ceftriaxone 2 g daily for four days until TTE was completed, which did not show myocardium inflammation. For the primary diagnosis of Lyme disease, the patient was given oral doxycycline 100 mg twice a day for 17 days on the day of discharge.

Follow-up and outcomes

**Figure 3:**
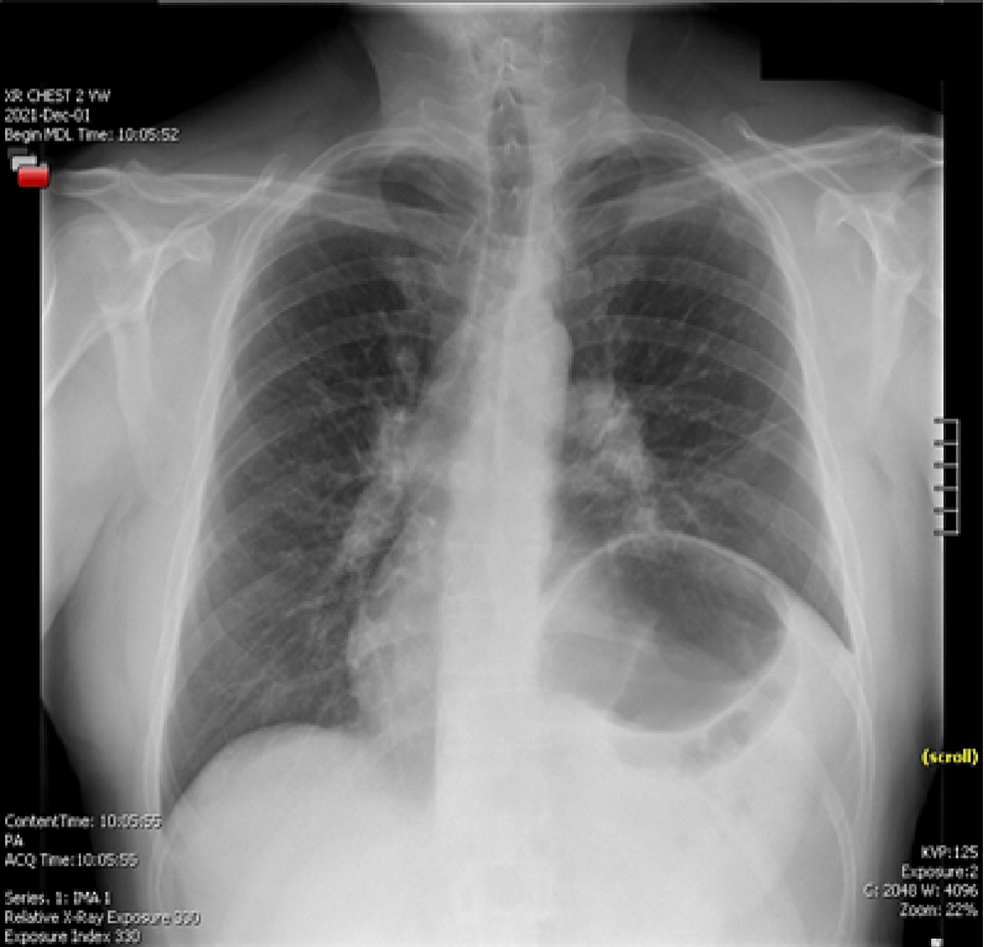
Chest X-ray showing no improvement in left hemidiaphragmatic paralysis after 64 days of initial treatment

The patient was seen in the outpatient setting eight days following discharge, at which time he stated adherence to antibiotics but was still experiencing shortness of breath with exertion. A chest x-ray (Figure 3) was repeated 64 days following discharge and showed persistent elevation of the left hemidiaphragm with left basilar atelectasis, unchanged from the prior chest X-ray. The patient was scheduled to follow up again in three months for a repeat chest X-ray for continuous monitoring.

## Discussion

Lyme disease is typically caused by the bacteria *Borrelia burgdorferi* and is the most common vector-borne disease in the United States. The northeast United States is considered an endemic area for Lyme disease [1] and our practice includes following the guidelines for antibiotic prophylaxis following a tick bite in addition to treatment guidelines once Lyme disease is diagnosed based on serology or physical exam findings such as erythema migrans. Following a tick bite, patients can be asymptomatic or develop mild cases of the disease, however, complications can result in cardiac and neurologic symptoms. With Lyme disease complications in mind, efforts should be taken to consider this in one’s differential diagnoses when clinically relevant. To date, there have been 16 cases reported on hemidiaphragmatic paralysis based on our literature search [3] with our case being the first presentation of atrial flutter likely secondary to hemidiaphragmatic paralysis causing structural disruption to the heart resulting in the arrhythmia. This patient converted to normal sinus rhythm while receiving diltiazem via drip and fortunately did not require further intervention for his cardiac arrhythmia. He has not reported any additional chest pain or palpitations since hospitalization, but he has experienced the persistence of dyspnea with activity. Reported cases in literature do show that hemidiaphragmatic paralysis can last up to a year, however, there is spontaneous resolution [3]. Serial monitoring with repeat chest X-rays can help if the patient continues to experience dyspnea due to hemidiaphragmatic paralysis to monitor progress and determine if an additional specialist referral is necessary.

Some strengths of this case include the thorough workup of the patient’s symptoms and prompt treatment. The patient had extensive diagnostic tests completed to help rule out other diagnoses considered on the differential. The information obtained from this case can help facilitate a deeper understanding of the complications seen with Lyme disease. A limitation of this case is that the patient moved out of the community making long-term follow-up challenging. However, his progress was monitored via virtual telemedicine. There is a lack of published research regarding this unique scenario. The patient’s atrial flutter spontaneously resolved on the diltiazem drip. However, had he remained in atrial flutter, the arrhythmia might have been difficult to manage given the lack of research on management in the setting of Lyme carditis and hemidiaphragmatic paralysis.

## Conclusions

In conclusion, it is important to consider different cardiac and neurologic conditions complicating Lyme disease in patients living in Lyme-endemic areas. This case shows that cardiac and neurologic complications can coexist. While rare, atrial tachyarrhythmias like atrial flutter can occur in the course of Lyme disease, and the presence of hemidiaphragmatic paralysis raises the question of its role in the genesis of the rhythm disturbance in this case. The clinical importance that Lyme disease complications can be extensive and even lead to long-term residual symptoms shows that it is possible for a patient with a history of Lyme disease to present with atrial flutter associated with hemidiaphragmatic paralysis. Complications of Lyme disease should be considered when cardiac or neurologic symptoms occur.
